# MetALD Molecular Signatures: What We Know, What We Lack, and How to Move Forward Through Integrated Multi-Omics

**DOI:** 10.3390/metabo16090608

**Published:** 2026-08-25

**Authors:** Miriam Longo, Marica Meroni, Erika Paolini, Paola Dongiovanni

**Affiliations:** Medicine and Metabolic Diseases, Fondazione IRCCS Ca’ Granda Ospedale Maggiore Policlinico, 20122 Milan, Italy; miriam.longo@policlinico.mi.it (M.L.); marica.meroni@policlinico.mi.it (M.M.); erika.paolini@policlinico.mi.it (E.P.)

**Keywords:** MetALD, lipidomics, metabolomics, biomarkers, artificial intelligence

## Abstract

**Highlights:**

**What are the main findings?**

- MetALD is emerging as a dynamic and heterogeneous subtype of the steatotic liver disease (SLD) spectrum, in which metabolic dysfunction and alcohol exposure do not simply coexist but rather drive different molecular trajectories.
- Metabolomic and lipidomic studies suggest that circulating multi-analyte panels can improve disease phenotyping by capturing alcohol-dominant and cardiometabolic-dominant signatures beyond conventional liver enzymes and self-reported alcohol intake.

**What are the implications of the main findings?**

- Dedicated MetALD cohorts with reliable assessment of metabolic risk and alcohol exposure are needed to validate reproducible omics-based signatures.
- The integration of multi-omics panels with clinical data, imaging, machine learning and digital/biomimetic twin approaches may support dynamic risk prediction and the personalized management of MASLD, MetALD and ALD.

**Abstract:**

With the advent of the new definition, fatty liver disorders have been reframed into metabolic dysfunction-associated steatotic liver disease (MASLD), alcohol-related liver disease (ALD), and the mixed phenotype referred to as MetALD (MASLD and increased alcohol intake). This change reflects the real-world clinical practice, where metabolic dysfunction and alcohol frequently coexist and synergize to increase risks of steatohepatitis, fibrosis, and hepatocellular carcinoma (HCC). While conventional non-invasive tests (NITs) remain the backbone of risk stratification, lipidomics and metabolomics can capture biological information on disease mechanisms and may improve early detection and prognosis. Here, we summarize the current evidence on circulating and tissue lipidomic and metabolomic signatures across MASLD, ALD and MetALD, discuss how the new definitions affect clinical risk assessment, and highlight recent studies which partially distinguish molecular fingerprints for mixed etiology disease.

## 1. Introduction

Steatotic liver disease (SLD) is among the leading causes of chronic liver disorder (CLD) worldwide, and its clinical spectrum is increasingly determined by the coexistence of cardiometabolic dysfunction (overweight/obesity, prediabetes/type 2 diabetes mellitus (T2DM), dyslipidemia, hypertension) and alcohol consumption. In routinary clinical practice, the overlap of these drivers may accelerate progression from simple steatosis to steatohepatitis, fibrosis, cirrhosis, and hepatocellular carcinoma (HCC) [1,2]. To reduce patients’ misclassification, the multisociety Delphi consensus has recently provided new definitions that explicitly capture both metabolic alterations and alcohol exposure. Within the SLD nomenclature, metabolic dysfunction-associated steatotic liver disease (MASLD) and alcohol-related liver disease (ALD) represent the two major ends. The former defined hepatic steatosis plus at least one cardiometabolic risk factor and alcohol intake ≤140 g/week in women or ≤210 g/week in men, while the latter refers to alcohol intake >350 g/week or >420 g/week in women and men, respectively. An intermediate category, referred to as MetALD (MASLD and increased alcohol intake), was also coined to identify patients fulfilling MASLD criteria plus moderate alcohol intake. However, the term MetALD was not conceived as a midpoint entity but rather as a heterogeneous condition in which the relative predominance of MASLD or ALD profiles may change longitudinally [3,4].

From a clinical standpoint, the updated SLD reclassification reshapes the diagnostic and prognostic evaluation. The pivotal issues remain the surveillance and management of patients at high risk of developing advanced fibrosis and portal hypertension using non-invasive tools. Current guidelines recommend an initial screening with blood-based fibrosis score (FIB-4) followed by elastography and/or serum fibrosis tests (e.g., ELF) with referral to hepatology when second-line assessment suggests advanced fibrosis or results are inconclusive [1,5]. Although these criteria are broadly generalizable across the CLDs, patients’ prognosis may be modulated by additional determinants in MASLD, ALD and MetALD categories. In MASLD, fibrosis staging is necessary, but it should be interpreted in the context of the underlying metabolic comorbidities, which may further amplify extrahepatic complications such as cardiovascular and chronic kidney disorders. In ALD, disease course is tightly influenced by alcohol ingestion to the extent that abstinence represents the strongest modifying factor. Conversely, continued drinking or episodes of alcohol-associated hepatitis increase the risk of decompensation and death, thus making the repeated medical examination of drinking patterns central to prognostic evaluation. MetALD adds a further layer of complexity since dysmetabolism and alcohol converge on partially shared injury pathways and may exert synergic effects on steatohepatitis, fibrosis and HCC risk [1,3,5,6,7]. As a result, predictive indicators derived from translational and clinical studies including MASLD or ALD patients may not be directly transferred to MetALD, supporting dedicated phenotyping and biomarker discovery approaches.

Recent longitudinal data further supports the need to move beyond the etiological classification [8]. In a UK Biobank analysis including 79,034 individuals with SLD, the incidence of major adverse liver outcomes, including ascites, cirrhosis, varices, liver failure and HCC, progressively increased from MASLD (1.91/1000 person-years) to MetALD (2.35/1000 person-years) and ALD (5.80/1000 person-years). Compared with MASLD, MetALD was associated with a 23% higher risk of liver-related events, whereas ALD showed the highest risk (HR 2.95). Conversely, MetALD and MASLD did not significantly differ in cardiovascular events or all-cause mortality, while both outcomes were more frequent in ALD [9,10]. These findings suggest that the current etiological categories identify different levels of liver risk, but they do not fully explain the variability in extrahepatic outcomes. Indeed, additional factors such as genetic susceptibility, body composition and cardiometabolic profile may further contribute to individual risk, supporting biological phenotyping beyond conventional SLD classification.

Against this background, lipidomics and metabolomics offer a high-resolution window into pathways that sit upstream of fibrosis, including de novo lipogenesis, lipoprotein trafficking, mitochondrial redox stress, bile acid signaling, and inflammatory lipid mediator biology. Unlike single analytes, -omics profiles can summarize coordinated shifts in lipid classes (e.g., triglyceride species composition, phospholipid remodeling, sphingolipids/ceramides) and metabolite networks (e.g., branched-chain amino acids, acylcarnitine, TCA intermediates, bile acids) that map onto disease mechanisms and may differ between MASLD-leaning and ALD-leaning phenotypes. Recent work also suggests that alcohol can imprint lipoprotein-associated lipidomes (such as the VLDL) and that plasma lipidomic patterns may help to discriminate MASLD from MetALD, highlighting a potential role for -omics in mixed exposure phenotyping [11].

Therefore, the central aim of this review is to debate whether current metabolomic and lipidomic findings support a distinguishable molecular signature that separates MetALD from MASLD and ALD rather than one reflecting its position as a midpoint disease. We also aim to discuss how the available evidence is consistently supported or still limited by human studies, since it derives almost entirely from single population-based cohorts, relies on self-reported alcohol intake, and describes a profile that largely tracks the degree of alcohol exposure and resolves into alcohol- and cardiometabolic-predominant subtypes. To this end, we first outline the epidemiology and the shared and distinct pathophysiology of the three entities; then, we discuss how alcohol exposure can be objectively assessed; and finally, we summarize the metabolomic and lipidomic signatures reported in MASLD, ALD and, specifically, MetALD, before addressing their integration and clinical translation.

## 2. MetALD Epidemiology

Within the SLD umbrella, the three subtypes carry different epidemiological weights. MASLD has a global prevalence estimated at around 30% of adults, which is rising in parallel with obesity and T2DM [12]. A meta-analysis of 72 studies including more than one million individuals reported a prevalence of 32.4%, with substantial ethnic variations and a clear temporal increase, from 26% in studies published up to 2005 to 38% in those published from 2016 onwards [13]. In contrast, ALD is less prevalent but disproportionately severe, since it accounts for the majority of cirrhosis and liver-related mortality worldwide, thus remaining a leading indication for liver transplantation in Europe and North America [14].

MetALD sits in between, and its prevalence has only recently begun to be estimated. Since the introduction of the term MetALD, most studies have assessed its prevalence in cohorts previously classified as ALD or MASLD or in large population datasets such as the UK Biobank. Reported prevalence ranged from 1.7% to 17.0%, depending on the population studied and SLD definition applied, with the lowest rates in the general population (1.7–4.5%), intermediate prevalence in primary care (5.6%), and the highest in alcohol-enriched cohorts (up to 17.0%). Diagnostic approaches have been heterogeneous, including CAP with different cutoffs, magnetic resonance imaging-proton density fat fraction (MRI-PDFF), ultrasound, or fatty liver index and, as expected, more sensitive techniques (MRI-PDFF or lower CAP thresholds) yielded higher prevalence estimates [15].

More recent population-based data from the UK Biobank have refined these estimates. In a cohort of 40,189 individuals undergoing liver MRI, 27.0% had SLD (PDFF ≥ 5%), and among those with SLD, 7.9% met MetALD criteria, corresponding to roughly 2% of the general population. MASLD accounted for the majority of SLD (89.0%), whereas ALD and at-risk MASH were less frequent. MetALD displayed a sex- and body mass index (BMI)-dependent pattern, being present in obese men and women but absent in non-obese women, suggesting that metabolic burden and alcohol intake interact differently across subgroups [15].

A recent systematic review and meta-analysis including 33 studies and over 7.5 million individuals further underscored the epidemiological relevance of MetALD. Across diverse settings, MetALD consistently emerged as a distinct SLD subtype with a non-negligible prevalence, particularly in populations with both metabolic risk factors and significant alcohol consumption. Although prevalence estimates vary according to region, methodology, and diagnostic criteria, the overall picture supports MetALD as a frequent overlap phenotype that nonetheless represents a recognizable condition within the SLD spectrum [16].

MetALD arises when hepatic steatosis, metabolic dysfunction, and alcohol intake above MASLD thresholds coexist [17]. Rather than a fixed midpoint between MASLD and ALD, it reflects a synergistic interaction in which metabolic stress heightens hepatocyte susceptibility to ethanol-derived injury, while alcohol amplifies lipotoxicity, oxidative stress, acetaldehyde-mediated toxicity, and mitochondrial dysfunction [17,18]. The fluidity of SLD subclasses over time, driven by changes in metabolic risk and alcohol consumption, has been recently described by Israelsen and colleagues [19]. In a prospective cohort, 36% of individuals classified as MetALD shifted toward MASLD (19%) or ALD (17%) at 6 months of follow-up. Similarly, 32% of those initially diagnosed with ALD moved toward either MetALD (21%) or MASLD (11%). Conversely, MASLD diagnosis was more stable with only 11% of individuals being reclassified over the same period. In addition, in a large population-based study including approximately 2.8 million individuals, changes in SLD subtype over time were associated with different liver-related risks. Transitions from MASLD or MetALD toward ALD were associated with an increased risk of liver-related events, whereas shifts from MetALD or ALD toward MASLD were associated with a lower risk. These findings emphasize the need for the repeated assessment of alcohol intake and cardiometabolic risk during follow-up, particularly in MetALD, to support timely and appropriate clinical management [20].

Beyond simple prevalence, newer data also characterize the cardiometabolic and clinical profile of MetALD. Plasma lipidomic analyses from the UK Biobank have identified that a specific, HDL-centric signature, characterized by enriched phospholipids and cholesterol within large and medium HDL subfractions, accurately discriminates MetALD from MASLD independently of self-reported alcohol intake [11].

Through a comprehensive proteomic and metabolomic analysis of the UK Biobank, Liu and colleagues demonstrated that MetALD segregated into an alcohol-dominant subtype and a cardiometabolic-dominant subtype, mirroring the biological architecture of ALD and MASLD, respectively. Notably, the alcohol-predominant signature was associated with significantly higher risks of advanced liver fibrosis, cirrhosis, and all-cause mortality [21].

Taken together, these epidemiological and phenotypic observations, coupled with emerging evidence of increased risk for advanced liver outcomes, reinforce that MetALD constitutes a clinically relevant SLD subtype, characterized by an intermediate non-benign risk profile and therefore requires dedicated diagnostic and therapeutic approaches [17].

## 3. MASLD and ALD Pathophysiology: Common Pathways Mechanisms and Disease-Specific Metabolic Cues

Although MASLD, ALD and MetALD are currently classified as distinct entities within the SLD nomenclature, their progression is sustained by partially overlapping mechanisms of liver injury. Hepatic lipid accumulation represents the earliest shared feature, but the biological processes leading to disease progression differ according to the predominant driver.

In MASLD, steatosis mainly results from excessive energy intake in the form of carbohydrates and fat which exceeds the oxidative and export capacity of the liver and promotes intrahepatic lipid accumulation. Hyperinsulinemia and sugars stimulate hepatic de novo lipogenesis (DNL) through *Sterol regulatory element-binding protein 1c* (*SREBP-1c*) and *Carbohydrate-responsive element-binding protein* (*ChREBP*) transcriptional programs, thus enhancing triglyceride (TGs) synthesis [22,23]. This process is further amplified by insulin resistance (IR) and adipose tissue lipolysis, which increases the release of free fatty acids (FFAs) into the circulation and enhances hepatic fatty acid uptake [1,22]. However, disease progression is not merely the consequence of TG storage. Changes in hepatic and circulating metabolomic and lipidomic profiles also contribute through the accumulation of lipotoxic species or the loss of protective lipids, including DAGs, free cholesterol, ceramides (Cers), oxidized lipids and phospholipids. These alterations may impair mitochondrial function and activate inflammatory signaling [22,24].

In addition, diets enriched in saturated fats, refined carbohydrates and poor in fibers may alter gut microbiota diversity and the production of bacterial-derived metabolites which have been associated with both hepatic lipid metabolism and cardiovascular risk. In particular, choline-betaine metabolism has gained attention in MASLD. The gut microbial conversion of choline into trimethylamine (TMA), which is oxidized to trimethylamine-N-oxide (TMAO), has been associated with MASLD onset and progression. Circulating TMAO levels have also been linked to MASH, particularly in individuals with obesity and T2DM [25,26]. Moreover, intestinal microbial species may modify the abundance of bile acid pools, short-chain fatty acids (SCFAs), tryptophan derivatives and branched-chain amino acids (BCAAs), thereby impacting on Farnesoid X Receptor/Takeda G-protein-coupled Receptor 5 (FXR/TGR5) pathways, which are essential for regulating glucose/lipid homeostasis, insulin sensitivity, intestinal integrity and systemic inflammation [27,28,29]. In parallel, obesity, IR and T2DM may further promote intestinal barrier dysfunction and low-grade inflammation, thus boosting bacterial by-products’ translocation into the portal circulation. These signals led to metabolic endotoxemia, activating hepatic immune and stellate cells (KCs: Kupffer cells; HSCs: hepatic stellate cells), which are involved in inflammatory injury and fibrogenic response [1,29].

Compared to MASLD, steatosis onset in ALD is a direct consequence of chronic alcohol intake. Indeed, ethanol is primarily metabolized by the *alcohol dehydrogenase* (*ADH*) enzyme and *cytochrome P450 2E1* (CYP2E1), which generate acetaldehyde, increase the NADH/NAD^+^ ratio and promote reactive oxygen species (ROS) production, thus unbalancing the redox status [30,31]. The excess of NADH, in turn, inhibits mitochondrial fatty acid β-oxidation through *AMP-activated protein kinase* (*AMPK*) and *peroxisome proliferator-activated receptor α* (*PPAR-α*) pathways and induces the synthesis of glycerol-3-phosphate (G3P), thereby enhancing DNL and facilitating fatty acid re-esterification into TGs [30,31]. Several studies reported that these molecular aberrancies, especially those related to impaired β-oxidation, are reflected by changes in acylcarnitines abundance in alcoholic patients. Gao et al. revealed that serum acetilcarnitine levels were higher in subjects presenting Mallory bodies at liver biopsy, a sign of hepatocyte damage, and their accumulation was markedly elevated in those who developed cirrhosis, organ failure or in deceased patients [32,33,34].

Similarly to MASLD, chronic alcohol intake is characterized by intestinal permeability, bacterial overgrowth and endotoxemia, which activate innate immunity and rapidly worsen liver function and clinical outcomes [7,30,35]. However, dysbiosis appears to induce a more specific gut-derived inflammatory and metabolic fingerprint in ALD, which is amenable to ethanol exposure. For instance, an elegant study from Duan et al. reported that cytolysin-positive *Enterococcus faecalis* was enriched in patients with alcohol-related hepatitis compared to non-alcoholic individuals and correlated with disease severity and mortality [36]. Moreover, the authors demonstrated that targeting hepatic cytolysin with bacteriophages strongly attenuates alcoholic hepatitis in humanized mice, supporting that the eradication of this specific bacterial strain might improve the clinical outcomes more than current treatments [37]. In an integrative analysis combining serum/fecal metabolomics and gut microbiota profile, it has been demonstrated that patients with advanced ALD were characterized by a broad remodeling of the serum and fecal metabolome, involving amino acids and related metabolites (glutamic acid, glutamine, 3-hydroxyanthranilic acid, kynurenic acid), nucleotide derivatives (2-O-methylcytidine, guanosine, inosine) and R-butyrylcarnitine correlating with specific bacterial species such as *Odoribacter splanchnicus* and *Coprococcus* sp. ART55-1. Overall, these findings indicate that alcohol-related dysbiosis contributes not only to endotoxemia but also to serum and fecal metabolic remodeling associated with ALD progression with circulating metabolites showing the strongest ability to discriminate advanced from milder disease forms [38].

Taken together, MASLD and ALD converge on several downstream mechanisms of liver injury, including lipid accumulation, mitochondrial dysfunction, oxidative stress and gut-derived inflammation, while differing in the relative contribution of metabolic and alcohol-related upstream drivers.

## 4. MetALD Pathophysiology: Does Dual Driver Exposure Translate into a Distinct Pathogenic Profile?

Given the mechanistic partial overlap in pathogenic pathways, recent translational studies have investigated whether the coexistence of metabolic dysfunction and alcohol exposure in MetALD generates a pathogenic profile that differs from either condition alone. Evidence suggests that the simultaneous exposure to these dual drivers does not result in a simple additive effect triggering a distinct, synergistic pathogenic profile characterized by reciprocal reinforcement.

At the hepatocellular level, the presence of IR alters the expression of key cytochrome enzymes, particularly upregulating CYP2E1. When alcohol is introduced, this enzymatic induction drastically accelerates ethanol oxidation, which in turn amplifies the generation of ROS and acetaldehyde [39]. This metabolic shift pushes the hepatocyte beyond its antioxidant capacity, causing severe mitochondrial structural dysfunction, oxidative and endoplasmic reticulum (ER) stress. These alterations lead to the activation of apoptotic pathways that far exceed the damage observed in metabolic conditions alone [40,41,42,43,44].

Furthermore, this dual-driver exposure establishes a unique multi-organ impairment that shapes a specific lipotoxic and inflammatory environment. In adipose tissue, metabolic dysregulation enhances lipolysis and the efflux of saturated fatty acids toward the liver. Concurrently, alcohol intake impairs the hepatic secretion of very-low-density lipoproteins (VLDL) and upregulates lipogenic transcription factors such as SREBP-1c and *Cluster of differentiation 36* (CD36) [45]. Rather than promoting steatosis, this metabolic and alcoholic synergy shifts the hepatic lipidome toward highly toxic lipid species, including Cers and DAGs. These lipotoxic intermediates directly disrupt the hepatic insulin signaling, worsening systemic IR and triggering apoptosis [44].

Finally, the distinct pathogenic profile of MetALD is defined by the disruption of the gut–liver axis and by the activation of innate immunity. Metabolic alterations and ethanol consumption act together altering the tight junctions of the intestinal epithelium and the bile acid composition, thus reshaping the gut microbiota [46]. This specific combination leads to a massive translocation of pathogen-associated molecular patterns (PAMPs), such as lipopolysaccharides (LPS), into the portal circulation. Within the hepatic parenchyma, these gut-derived PAMPs activate the liver’s innate immunity through *Toll-like receptors 4 and 9* (TLR4, TLR9) pathways [47]. Finally, this molecular crosstalk triggers the *NOD-like receptor family pyrin domain containing 3* (NLRP3) inflammasome and drives the transdifferentiation of quiescent HSCs into collagen-producing myofibroblasts, accelerating inflammation and fibrogenesis [43,44].

Regarding disease severity, it is not clear whether fibrosis prevalence in MetALD differs from that in MASLD and ALD. In a Korean study, the assessment of fibrosis through MRI revealed higher values in MetALD patients compared to MASLD and ALD groups [48]. Similar results were found by Choe et al. in another Korean cohort where the hazard ratios (HRs) for advanced liver fibrosis in patients with MASLD, MetALD, and ALD were 1.39 [95% confidence interval (CI): 1.25–1.55; *p* < 0.001], 1.75 (95%CI: 1.38–2.23; *p* < 0.001), and 2.00 (95%CI: 1.30–3.07; *p* = 0.002), respectively, thus suggesting that MetALD patients have an intermediate risk of developing advanced liver fibrosis [49]. Opposite findings were described by Ciardullo et al. and by Yang et al. in United States studies where the prevalence of liver fibrosis was comparable in MASLD and MetALD patients [50,51].

In a retrospective cohort study of HCC patients (n = 577) conducted in Berlin between 2010 and 2020, the etiologies were MASLD (n = 158; 27.3%), MetALD (n = 51; 8.8%), and ALD (n = 68, 11.7%) [32]. Regarding HCC progression induced by SLD, a Korean study from 2009 to 2010 reported that it grew from MASLD (0.6%) and MetALD (0.92%) to ALD (1.26%) in a stepwise manner [52,53]. Similar results were reported by Kim et al., who demonstrated that the incidence of decompensated cirrhosis rose from MASLD (1.1%) to MetALD (1.3%) and ALD (2.2%); and the corresponding subdistribution hazard ratios (HRs) were 1.45 (95%CI: 1.29–1.62; *p* < 0.001), 1.77 (95%CI: 1.47–2.14; *p* < 0.001), and 2.24 (95%CI: 1.74–2.89; *p* < 0.001). These data suggest that MetALD may be associated with an intermediate risk of liver disease severity in the SLD spectrum. However, discrepancies across studies may occur and are possibly ascribable to the severity of the underlying metabolic risk factor, the amount of alcohol consumption, the genetic background and the non-invasive or histological assessment of liver damage.

## 5. From Self-Reports to Biomarkers: Redefining How We Measure Alcohol Intake

The MetALD definition relies on self-reported alcohol intake, including the amount consumed, drinking patterns (daily versus binge drinking), and duration of exposure, although gathering a reliable consumption history can be challenging. The definition of alcohol consumption categories has been established by the National Institute of Alcohol Abuse and Alcoholism (NIAAA) in standard, moderate, heavy and binge drinking [2]. However, it is difficult to standardize these categories, since both the definition of a standard drink (i.e., 8 g of pure alcohol in the UK, 10 g in Europe, 14 g in the USA and Latin America, and 23 g in Japan) as well as serving sizes (1 standard drink equals 296 mL of beer, 133 mL of wine and 30 mL of distilled spirits in the UK but 356 mL of beer, 148 mL of wine, and 44.4 mL of distilled spirits in the US) vary across countries [54].

In addition, some authors have proposed the term “glass-year” to describe the average number of glasses of alcohol a person has drunk over time, analogous to the smoking-related pack-year to quantify the smoking status. Thus, one glass-year is defined as one glass of alcohol daily for 1 year, although a glass of alcohol should be universally defined [55].

Self-reported questionnaires, such as the Alcohol Use Disorders Identification Test (AUDIT) and its shorter version, AUDIT-C, are currently used to estimate alcohol consumption [2,56]. The AUDIT is a 10-item questionnaire assessing alcohol consumption, drinking behavior, and alcohol-related consequences with scores ranging from 0 to 40. An AUDIT score ≥ 8 identifies hazardous or harmful drinking with approximately 92% sensitivity [57]. The AUDIT-C is a three-item screening tool derived from the full AUDIT questionnaire, which is specifically designed to assess hazardous drinking, showing a cutoff of ≥3 for women and ≥4 for men. Despite their utility, AUDIT-based tools have lower sensitivity for detecting alcohol use compared with some alcohol biomarkers, which should be incorporated into routine clinical practice for a more accurate assessment of recent alcohol consumption. Indirect markers such as gamma-glutamyl transpeptidase (GGT) and transaminases as well as carbohydrate-deficient transferrin (CDT) are commonly used by clinicians to assess alcohol intake, although they primarily reflect sustained abuse and can be affected by confounding factors.

Among direct alcohol biomarkers, phosphatidylethanol (PEth) has emerged as the preferred one, since its blood concentrations correlate strongly with alcohol intake and it offers the longest detection window (approximatively 3–4 weeks) [18]. PEth is a phospholipid formed in blood red cell membranes where the absence of the degradative enzyme phosphatidylcholine-specific phospholipase C (PC-PLC) results in its accumulation to measurable levels. In United States commercial laboratories, a cutoff of 20 ng/mL indicates significant alcohol use. However, a recent study employing a continuous transdermal alcohol sensor as the reference standard identified 18.5 ng/mL as the optimal PEth threshold for detecting heavy drinking, which is defined by the NIAAA as >7 drinks per week or >3 drinks on any day for women and >14 drinks per week or >4 drinks on any day for men [58]. Higher PEth concentrations provide additional discriminatory value. Several studies have demonstrated that concentrations from 200 ng/mL up to 60 g/day indicate daily drinking and binge drinking, respectively, which represents the upper threshold for the MetALD definition. Similarly, PEth > 200 ng/mL is consistent with alcohol consumption >60 g/day (men) proposed to identify ALD. Therefore, PEth could be useful to distinguish the SLD subtypes, although further studies are required to establish its diagnostic performance [59,60].

As mentioned above, PEth concentrations should not be interpreted as a direct quantitative estimate of alcohol intake. In a longitudinal study of individuals undergoing treatment for reduced drinking, PEth levels correlated well with past weeks’ alcohol intake, albeit with a large inter-individual scatter, thus indicating that a single PEth measurement may increase the risk of misclassification between moderate and heavy drinking [61]. Consistently, a marked difference among subjects was observed in PEth clearance, its half-life or homologues (such as PEth 16:0/18:1, PEth 16:0/18:2) during alcohol abstinence [62]. More recently, Stark et al. showed that PEth formation rates also vary between individuals exposed to comparable ethanol concentrations, supporting the use of serial measurements to monitor changes in alcohol exposure within the same patient [63]. Despite these limitations, PEth remains particularly useful when interpreted together with self-reported alcohol intake, as it may identify alcohol exposure that would otherwise be missed and consequently improve SLD classification.

In a prospective study including 391 community-dwelling adults with overweight/obesity and SLD, Tavaglione and colleagues reported substantial misclassification when relying solely on self-reported intake. They found that 16% of individuals were misclassified as having MASLD and 29% as having MetALD [60]. Moreover, the integration of PEth concentration to self-reported alcohol consumption resulted in a 4-fold and 3-fold increase in MetALD and ALD diagnosis, respectively. Based on these findings, the same authors recently proposed the MetALD-ALD Prediction Index (MAPI), using PEth-based classification as the reference standard for identifying excessive alcohol exposure in individuals with SLD and combining it with sex, mean corpuscular volume, GGT, HDL cholesterol and HbA1c. The MAPI score was developed in a cohort of 503 individuals with overweight/obesity and SLD and externally validated 1777 individuals [64]. The clinical relevance of an objective assessment of alcohol exposure was further supported by recent studies showing a substantial reclassification of SLD subtypes after PEth measurement. Consistently, Torp et al. found that 39% of individuals classified as MASLD according to self-reported alcohol intake had PEth concentrations compatible with MetALD or ALD [65]. Hence, an inaccurate assessment of alcohol exposure may affect not only clinical classification but also lead to the inclusion of biologically heterogeneous patients within the same diagnostic group, affecting biomarker discovery and validation.

In summary, a standardized approach to quantifying alcohol intake is essential for accurate MetALD classification. Diagnosis should be supported by the assessment of metabolic dysfunction (≥1 of the five criteria), a reliable self-reported alcohol history with collateral information from family or caregivers, and, when appropriate, a confirmation of alcohol intake through serial PEth measurements (20–200 ng/mL) over a 3-month period. This combined strategy aids MetALD diagnosis and patient management.

## 6. Metabolomics and Lipidomics Panels: From Single Biomarkers to Molecular Phenotyping

The search for biomarkers in metabolic liver disorders has traditionally relied on single circulating analytes (basal glycemia, insulin, bilirubin, albumin etc.) or conventional parameters for calculating non-invasive scores of fibrosis based on aminotransferases (ALT, AST), GGT, lipids, bilirubin, albumin, platelets (FIB-4, APRI, ELF etc.). As above mentioned, although these markers remain useful in clinical practice, they provide only a partial representation of metabolic derangement arising from the convergence of multiple systemic, hepatic and inter-organ interplay, often lacking sufficient specificity to distinguish simple steatosis from MASLD and ALD progressive forms [1,5].

With the advent of -omics technologies, biomarker discovery has progressively shifted from the measurement of isolated-to-panels of metabolites that together provide a broader sight of disease-related changes. Among the different omics layers (such as genomics, transcriptomics, proteomics), metabolomics and lipidomics are particularly attractive in this field because they directly measure small molecules and lipid species closer to the functional phenotype, potentially reflecting the downstream consequences of nutrient intake, alcohol exposure, IR, mitochondrial activity, gut microbiota metabolism and organs’ crosstalk [66,67].

Two analytical platforms are mainly exploited: (a) the nuclear magnetic resonance (NMR) spectroscopy, which is the most reproducible and quantitative method across population studies [68] as it requires minimal sample preparation, and (b) mass spectrometry (MS) coupled to liquid or gas chromatography, which covers a far wider portion of the lipidome and can resolve individual species. This is an important point for several lipid classes, such as Cers, which can be conjugated with different acyl chains. However, MS is more sensitive to pre-analytical and batch effects, and it is harder to standardize across laboratories [67].

The main advantage of these analyses is their applications in non-invasive matrices, as serum or plasma, making them utilizable on large scales, including adults, pediatrics as well as healthy subjects for case-control studies. This aspect may be particularly relevant since, as previously described, MASLD and ALD show great overlapping features, also in terms of circulating effectors (i.e., acylcarnitines), which may not be disease-specific when considered individually [32,69].

Therefore, moving from a single marker to a panel may be more informative for several reasons. The first one is that the same molecule can move in opposite directions depending on the driver. Ceramides, for instance, accumulate in MASLD and correlate with IR, while their abundance together with that of sphingomyelins reduces in alcohol-related fibrosis [70,71]. Secondly, patients with the same amount of liver fat do not always show the same molecular profile, because steatosis is a shared endpoint reached through different routes. For instance, in 125 liver biopsies, hepatic ceramide enrichment is still associated with IR but not with steatosis driven by the presence of PNPLA3 I148M variant [70], thus suggesting that genetic background contributes to this variability.

## 7. Lipidomic and Metabolomic Signatures in MASLD and ALD

To answer whether MetALD is molecularly distinct from MASLD and ALD, we first require defining the profiles of the two conditions from which it arises. For this reason, here we summarize the metabolomic and lipidomic changes established in MASLD and ALD separately. As described above, the two pathologies converge on hepatic lipid accumulation, but their molecular signatures, in terms of the species that accumulate or decline along the course of disease, may not overlap.

As described before, MASLD and ALD converge on hepatic lipid accumulation, but the molecular signatures, with species that accumulate or disappear along the course of disease, may not overlap. Here, we briefly summarize the principal findings of metabolomic and lipidomic studies in the two separate conditions.

The first systematic analysis of liver biopsies across the MASLD spectrum (previously known as non-alcoholic fatty liver disease, NAFLD) showed that the difference between simple steatosis and steatohepatitis (NASH) was qualitative rather than quantitative. Beyond the expected accumulation in TGs, DAGs and free cholesterols, disease progression was accompanied by the depletion of long-chain polyunsaturated fatty acids (PUFAs) and a shift in the PC/phosphatidylethanolamine (PE) ratio, which affects membrane stability and VLDL export [72]. In plasma, the transition from NAFL to NASH was marked by an increase in arachidonic acid-derived eicosanoids (5-, 8- and 15-HETE), 11-HETE and oxidized lipids, which mainly tracked the inflammatory status and oxidative stress rather than hepatic fat fraction [73,74]. This evidence has recently been confirmed in 301 patients with biopsy-proven MASLD from the NASH Clinical Research Network cohort, whereby a panel of 15 bioactive lipids, mostly eicosanoids, discriminated MASLD from healthy controls with an AUROC of 0.99 [75]. Metabolome analysis comparing the plasmatic profile of 44 NAFLD patients with obesity (NAFLD-Ob) or without obesity (NAFLD-NO) against non-obese individuals revealed that most amino acids were increased only in NAFLD-Ob, while in NAFLD-NO subjects, only alanine, glutamate, isoleucine and valine rose above controls, suggesting that the amino acid signature was largely driven by obesity and IR [70,76]. In the same cohort, however, the glutamate–serine–glycine (GSG) index correlated with hepatic IR and with liver enzymes, independently of BMI, and it was proposed as a marker of disease severity [76].

These observations have been further translated into candidate diagnostic tools. Indeed, a serum panel based on TG species discriminated NAFLD from normal liver with an AUC equal to 0.88 and NASH from NAFL with a 0.79 AUC in two independent NAFLD cohorts [77]. The Metabolomics-Advanced Steatohepatitis Fibrosis (MASEF) score, combining 12 lipid species with BMI, AST and ALT, identified at-risk MASH with AUROC values of 0.76 and 0.79 in discovery (n = 790) and validation (n = 565) cohorts, which was a performance comparable to that of transient elastography but obtainable from a blood sample [78].

In an ALD study including 315 biopsied patients with early diseases, the researchers paired liver and plasma samples and found that most sphingomyelins, Cers and PC were co-downregulated in both compartments, and their abundance fell as fibrosis progressed. Sphingomyelins showed the strongest inverse correlation with fibrosis and inflammation, and low levels were predictive of future liver-related events to the extent that Mendelian randomization supported a causal relationship between ALD worsening and sphingolipid depletion [71].

As above-mentioned, the alcohol-related metabolome carries a distinct mitochondrial fingerprint due to the direct impact of ethanol on increases in the NADH/NAD^+^ ratio and harmful effects on β-oxidation, causing acylcarnitines accumulation in the liver and circulation [32]. This molecular feature has recently gained prognostic weight. In patients with severe alcohol-related hepatitis, untargeted lipidomics identified a glycerophospholipid signature discriminating alcohol-related hepatitis from decompensated cirrhosis (AUROC 0.87–0.88), while short-chain acylcarnitines such as CAR (2:0) and CAR (16:1) were independently associated with 90-day mortality and correlated with MELD score, pro-inflammatory cytokines and hepatocyte ballooning [32].

## 8. Multi-Omics Evidence Defining the Molecular Profile of MetALD

In recent years, omics technologies have emerged as powerful tools for unraveling the biological complexity of MetALD. Recently, Liu et al. integrated proteomic and metabolomic profiling to demonstrate that MetALD is a heterogeneous entity rather than a simple overlap between MASLD and ALD. Multi-omics analyses revealed two phenotypes driven by differential pathway activation: an alcohol-dominant subtype characterized by neutrophil-mediated inflammatory cascades, oxidative stress responses, and extracellular matrix remodeling, and a cardiometabolic-dominant subtype marked by disruptions in lipid handling, mitochondrial β-oxidation, bile acid synthesis, and steroid hormone metabolism. Proteomic signatures highlighted complement activation, acute-phase reactants, and cytoskeletal remodeling, while metabolomic profiles identified alterations in acylcarnitines, sphingolipids, and bile acid derivatives that may contribute to differentiate MetALD from MASLD and ALD. Importantly, the proteomic-based subtyping identified an alcohol-predominant MetALD group characterized by higher fibrosis burden as well as increased risks of cirrhosis and mortality compared with the cardiometabolic-predominant subtype, thus supporting their prognostic utility for risk stratification [21].

Wolkmar et al. combined the NMR-based metabolomic as a ‘liquid biopsy’ with circulating HCC-associated biomarkers and MELD-based risk assessment across MetALD, MASLD, ALD and healthy controls. The authors showed that specific metabolite patterns, including changes in acetoacetate, 3-hydroxybutyrate, apolipoprotein A1 (APOA1), and PC, identify MetALD patients at higher risk of liver disease progression, particularly among women. The study highlighted a recognizable systemic metabolic signature and biologically relevant sex-specific differences, thus proposing the use of metabolomic panels for early diagnosis and risk stratification, moving beyond traditional liver enzymes and self-reported alcohol intake [79].

In a large population-based cohort, lipidomics and metabolomics have been used to distinguish MASLD, MetALD, and ALD across 40,534 individuals in the UK Biobank project. The study identified HDL-centric lipidomic markers, ketone bodies (acetoacetate, 3-hydroxybutyrate), and PC as key discriminators of MetALD. Mendelian randomization analyses demonstrated that alcohol consumption plays a causal role in modulating these metabolites. Collectively, these findings suggest that MetALD may be distinguished from MASLD and ALD for its own molecular footprint at the population level, which largely reflects the relative contribution of alcohol exposure and cardiometabolic dysfunction, and that omics can bridge epidemiology and mechanistic biology [68].

A recent meta-analysis aggregating clinical and laboratory data from multiple cohorts included in observational studies has further defined the metabolic and biochemical profile of MetALD. Compared to MASLD, MetALD is associated with higher liver enzymes (AST, ALT, GGT) and higher blood pressure but lower BMI and HbA1c, suggesting an “alcohol-weighted” phenotype that nonetheless retains substantial metabolic dysfunction. Conversely, liver enzymes are lower in MetALD than in ALD, whereas many metabolic parameters are comparable, reinforcing the concept that MetALD has a specific laboratory and metabolic signature compared to MASLD, whereas it differs less from ALD [16].

Overall, current multi-omics data support that MetALD is associated with recognizable molecular and metabolic patterns, which may discriminate it from MASLD and, to a lesser extent, from ALD, and that multi-analyte panels including proteins, metabolites, lipid species can improve phenotyping and risk stratification beyond conventional liver enzymes and self-reported alcohol intake. However, the available evidence should be carefully interpreted, since the circulating signals (lipoproteins, ketone bodies, acylcarnitines and bile acid by-product) do not yet establish a unique MetALD-specific molecular signature. Indeed, most studies have been conducted in population-based or pre-existing cohorts rather than prospectively recruited MetALD populations, and several of the reported biomarkers appear to reflect the relative contribution of alcohol exposure and cardiometabolic dysfunction. Consistently, molecular analyses tend to resolve MetALD into alcohol-predominant and cardiometabolic-predominant subtypes rather than a single independent profile. Moreover, despite the consistency of data across different analytical methods, determining the stability, prognostic relevance, and clinical utility of these signatures requires replication in independent and well-defined MetALD cohorts, integration with liver tissue-omics, and longitudinal validation. Table 1 summarizes the metabolome/lipidome signatures in MASLD, ALD and MetALD.

## 9. Integration of Multi-Omics Panels and Clinical Implications

Metabolomic and lipidomic panels have shown promising diagnostic and prognostic performance across different SLD etiologies, but their clinical applicability remains limited, particularly in MetALD, where dedicated validation studies are still lacking.

At present, the use of metabolomics and lipidomics data may complement existing non-invasive tests (NITs). The MASEF score represents the clearest example, since it combines a limited number of lipid species with routine parameters (BMI, AST, ALT) and, when incorporated into a FIB-4, achieved a performance comparable to that of FIB4-liver stiffness measurement (LSM), thus offering an alternative where elastography is unavailable. However, MASEF was developed and validated in MASLD cohorts, and its diagnostic performance has not yet been established in MetALD [78]. More generally, the clinical application of these approaches presents several constraints related to etiological classification and prospective validation.

As concerns the former, it has been discussed that self-reported alcohol intake or clinical history cannot reliably assign a patient with SLD to a metabolic-dominant or an alcohol-dominant profile, as demonstrated by the substantial rate of reclassification observed when PEth is included for patients’ evaluation [88]. In this context, molecular profiling may provide additional prognostic information within MetALD [21]. Nonetheless, these molecular subtypes have so far been identified in a limited number of cohorts and require external validation before their prognostic value can be translated into clinical practice. Beyond prognosis, identifying the predominant disease driver may also become relevant for treatment, since MetALD requires interventions addressing both alcohol exposure and cardiometabolic dysfunction. A recent randomized, double-blind, placebo-controlled trial (NCT05895643), lasting 26 weeks in patients with alcohol use disorder and obesity, showed that semaglutide significantly reduced heavy drinking days (−41.1 percentage points from baseline compared to placebo). These data support the potential of therapies acting on both metabolic and alcohol-related components, although this approach has not yet been tested specifically in MetALD cohorts [89].

Regarding the longitudinal studies, lipidomic and metabolomic signatures are dynamic and may in principle track abstinence, weight loss or the response to emerging pharmacological treatments requiring future reassessment and therefore increasing time for both analytical findings and costs. The same heterogeneity should also be considered in the design of MetALD clinical trials. A recent proposal recommends stratification according to fibrosis stage and drinking patterns together with the inclusion of alcohol-reduction and cardiometabolic endpoints in addition to liver-related outcomes [90].

In this scenario, combining lipidomics and metabolomics panels with imaging techniques (i.e., MRI), genetics, and other omics such as gut microbiota or proteomics becomes necessary to increase the biological resolution and improve diagnostic accuracy, but it also generates datasets involving hundreds of correlated features and interactions which cannot be handled through conventional statistics. Machine learning (ML) and artificial intelligence (AI) approaches are increasingly used to integrate large and heterogeneous clinical and multi-omics datasets and to develop diagnostic and prognostic models. However, their application in MetALD remains largely investigational and requires prospective and external validation before routine clinical implementation.

In the LITMUS cohort, including 966 patients with biopsy-proven MASLD, the authors built gradient boosting models with 35 predictors, comprising routine clinical variables and protein markers of fibrogenesis and apoptosis (ELF test, PRO-C3, PRO-C4, PRO-C6, cytokeratin-18) to predict steatosis, inflammation and ballooning separately and then combining the three models. The second strategy performed considerably better, raising the AUC for at-risk MASH from 0.65 to 0.83 even though the presence of protein markers did not further improve the model. Conversely, their addition for fibrosis staging resulted in an improvement of the AUC [91]. More recently, Stefanakis et al. developed lightweight ML models to identify biopsy-proven MASH with F2–F3 fibrosis in 443 individuals recruited across three countries and different clinical settings. Routine clinical variables, including aminotransferases, components of metabolic syndrome, BMI and two circulating metabolites (3-ureidopropionate, α-ketoglutarate), were integrated into the models. The model including 3-ureidopropionate achieved an AUC of 0.89, which increased to 0.91 after the addition of α-ketoglutarate. The ML-based models outperformed conventional NITs and maintained their performance when tested across independent cohorts, becoming of interest for patients’ eligibility to Resmetirom administration or phase III MASH trials [92].

Although such approaches aid the identification of at-risk MASH, they remain cross-sectional and cannot anticipate how an individual patient will evolve over time or respond to a given treatment. Beyond these data-driven strategies, digital and biomimetic twins represent a more experimental step toward personalized disease modeling and should currently be considered as future research tools rather than established clinical applications. Among them, patient digital twin (PDT) features computational representations of the individual patient built on clinical parameters (clinomics) and multi-omics data, which may be progressively updated to simulate changes in health status or responses to interventions. Initial clinical evidence derives mainly from studies enrolling patients with dysmetabolic features with potential liver involvement. In a one-year randomized controlled trial including 319 patients with T2DM, a PDT based on continuous glucose monitoring, wearable sensors, food logs and clinical data was used to provide personalized nutritional recommendations through ML algorithms, and it improved MASLD management compared to standard care [93]. However, liver-related outcomes were secondary endpoints, and this approach has not been validated for fibrosis progression or the prediction of clinical outcomes in dedicated MASLD or MetALD cohorts.

Liver-specific virtual twin applications have only recently started through initiatives such as the ARTEMIS project launched in 2024. ARTESMIS includes a European observational cohort and coupled computational models of the liver and the heart, integrating clinical, imaging and molecular data, with the aim of building a clinical decision support system able to predict disease progression and cardiovascular events. Mechanistic and AI-driven models are being validated in four clinical scenarios, considering fibrosis staging, cardiovascular complications, transjugular intrahepatic portosystemic shunt and liver transplantation (NCT07430501). Although these approaches may ultimately allow longitudinal patient-specific modeling, their ability to improve risk prediction or guide therapeutic decisions remains to be demonstrated prospectively.

At an earlier stage of development, patient biomimetic twins (PBTs) have been proposed as an experimental counterpart to computational PDTs. Miedel et al. recently proposed to couple each PDT with PBT, consisting of organoids or induced pluripotent stem cells (iPSCs) derived from the same individual and differentiated within microfluidic systems (such as liver-on-a-chip). With this approach, the in silico predictions originated from PDT are followed by experimental validation with PBT before being applied to clinical practice. In addition, the experimental results may be then re-used to refine the computational model with the goal of improving precision medicine procedures [94]. However, this integrated PDT-PBT approach has not yet undergone clinical validation and should therefore be regarded as a future precision-medicine strategy.

**Figure 1:**
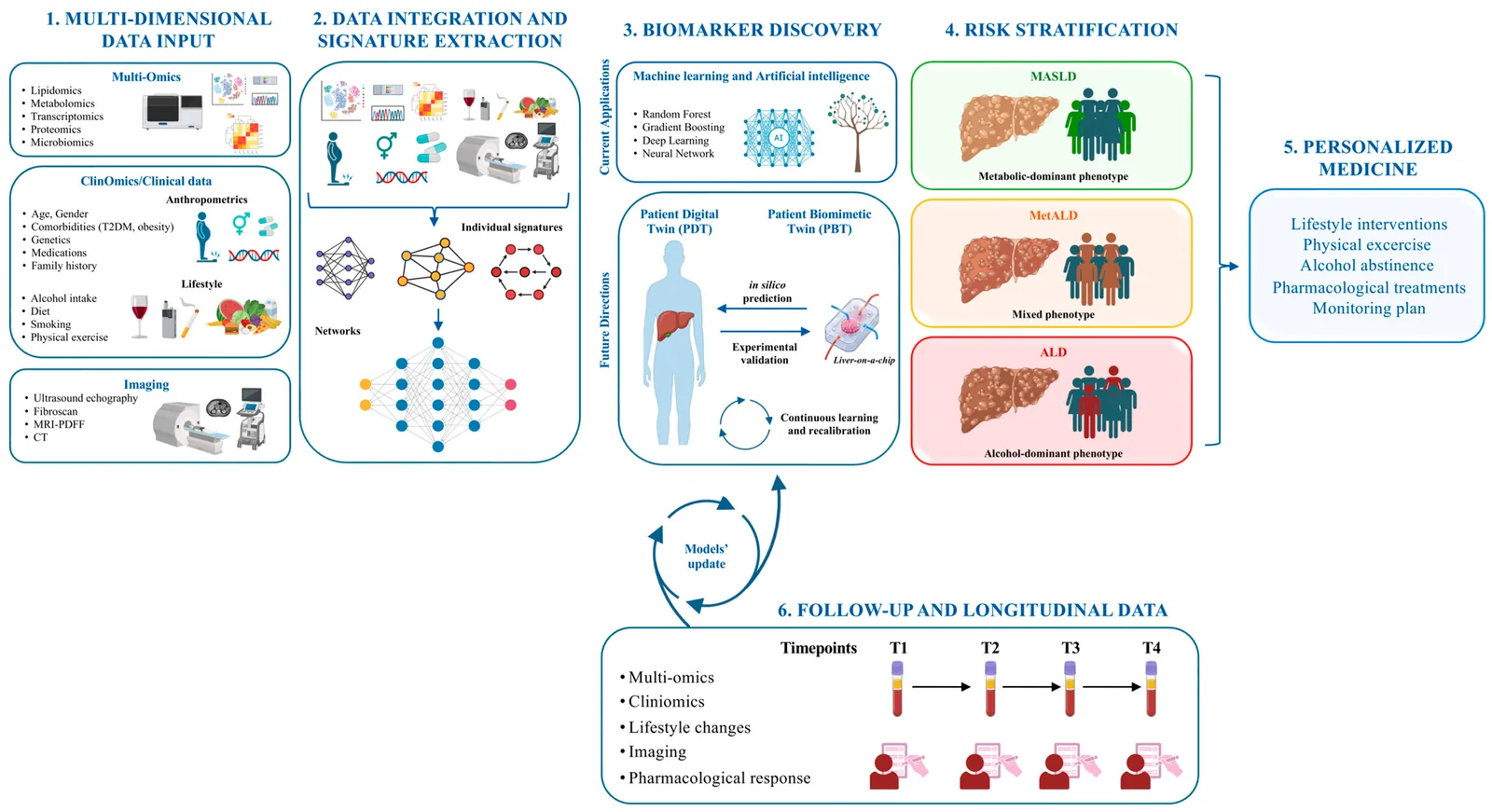
Schematic workflow for the use of metabolomics, lipidomics and other omics datasets in steatotic liver disease (SLD). Multi-dimensional data, including circulating and tissue -omics, clinical and demographic variables, lifestyle exposures, genetic background and imaging, can be integrated to extract individual molecular signatures and pathway networks. These data may then support biomarker discovery through machine learning (ML) and artificial intelligence (AI) approaches, representing the current or near-term translational applications of multi-omics integration. Moreover, patient digital twins (PDTs) and patient biomimetic twins (PBTs), including patient-derived organoids or liver-on-a-chip systems, should currently be regarded as future or experimental tools. In this setting, PDTs simulate disease trajectories and predict individual risk, while PBTs experimentally validate the in silico predictions. The resulting information could improve risk stratification across metabolic dysfunction-associated steatotic liver disease (MASLD), alcohol-related liver disease (ALD) and MASLD and increased alcohol intake (MetALD) helping to distinguish metabolic-dominant, mixed and alcohol-dominant phenotypes. Longitudinal follow-up, including repeated multi-omics profiling, clinical reassessment, imaging, lifestyle changes and pharmacological response, may progressively update prediction models and support personalized management.

Overall, the road toward clinical application is still long. Omic analyses are expensive, technically demanding and not easily compatible with routine outpatient workflows. The use of different analytical methods require standardization before being applied to hospitals, and data interpretation needs elevated computational skills. In addition, with the advent of new definitions, dedicated MetALD cohorts are still lacking, and the available panels have been built in populations in whom alcohol intake was either excluded or poorly quantified. Therefore, the implementation of these platforms will require time, standardized clinical data collection and prospective validation in well-characterized cohorts. A schematic representation of multi-omics application for clinical risk stratification is reported in Figure 1.

## 10. Conclusions and Future Perspectives

Concerning the central question of this review, whether MetALD displays a distinguishable metabolomic and lipidomic signature, the currently available evidence provides only a partial and preliminary answer. MetALD is increasingly recognized as a distinct condition within the SLD spectrum, and interest in defining its molecular and metabolic profile is rapidly growing. Omics-based investigations specifically designed to characterize MetALD remain limited, and most molecular insights still derive from MASLD and ALD research rather than from dedicated MetALD cohorts. As a result, current knowledge reflects a combination of direct evidence, few circulating omics signatures identified in MetALD, and indirect evidence inferred from the biology of metabolic dysfunction and ethanol metabolism. This imbalance highlights that the available data, while suggestive of a distinct molecular profile, are insufficient to define a stable and reproducible MetALD signature.

To achieve this objective, several gaps need to be addressed. First, dedicated omics studies must be conducted in well-phenotyped MetALD cohorts, with standardized definitions of metabolic dysfunction and alcohol exposure, and involve the systematic use of direct alcohol biomarkers such as PEth. Second, circulating lipidomics and metabolomics should be complemented with liver tissue omics, including transcriptomics, proteomics, and hepatic metabolomics/lipidomics to capture intrahepatic heterogeneity. Third, longitudinal designs are essential to assess the stability and reversibility of MetALD signatures in response to changes in drinking patterns, weight loss, metabolic control or pharmacological treatment. Fourth, multi-omics data should be integrated with clinical variables, imaging (MRI-PDFF, elastography), and NITs within unified analytical approaches, leveraging machine learning to refine MetALD subtyping and to derive predictive models. Fifth, clinical translation will require the harmonization of pre-analytical procedures, including sample collection, processing and storage, as well as standardized analytical workflows and the assessment of inter-laboratory and inter-platform reproducibility. Candidate panels will also require external validation in independent cohorts, the assessment of cost-effectiveness, and the demonstration of incremental diagnostic or prognostic value over currently available NITs before routine clinical implementation. Through these coordinated efforts, it will be possible to move from preliminary molecular patterns to validated, clinically useful signatures that can guide diagnosis, risk stratification, and the personalized management of MetALD patients.

## Figures and Tables

**Table 1:** Schematic representation of metabolome/lipidome signatures in MASLD, ALD and MetALD.

Species	MASLD	ALD	MetALD	Biological/Clinical Relevance	Ref.
***Neutral lipids***	↑ TGs, ↑ DAGs, ↑ free cholesterol	↑ TGs, ↑ FFAs	↑ DAGs *	Steatosis, lipotoxicity	[72,80]
***Ceramides/*** ***Sphingomyelins***	↑ Cer, **↕** SM	↓ Cer, ↓ SM	↑ Cer *	IR, fibrosis/prognosis	[70,71,81,82]
***Phosphatidylcholines/*** ***Phosphatidylethanolamines***	**↕** PC, ↓ PC/PE ratio	↓ PC	↑ PC vs. MASLD **^†^**	Membrane remodeling, VLDL export/ disease severity	[11,71,72,79,83,84]
***Acylcarnitines***	↑	↑	**—**	β-oxidation/prognosis	[32,33,34,69,85,86,87]
***HDL/*** ***HDL-associated lipids***	↓ vs. MetALD	↑ Large HDL particles, ↑ HDL cholesterol, ↑ HDL phospholipids	↑ Large HDL particles **^†^**, ↑ HDL cholesterol **^†^**, ↑ HDL phospholipids **^†^**,	Lipoprotein metabolism/ MetALD phenotyping	[11]
***Ketone bodies***	↓	↑ Acetoacetate, ↑ 3-hydroxybutyrate, ↑ acetone	↑ Acetoacetate **^†^**, ↑ 3-hydroxybutyrate **^†^**	Redox balance, energy metabolism/MetALD phenotyping	[11,79]
***Eicosanoids/*** ***Oxidized lipids***	↑ 5-, 8-, 15-HETE, ↑ 11-HETE	↑ 5-HETE, ↑ 20-HETE ↑ 9-, 13-HODE	**—**	Steatosis, inflammation, oxidative stress	[73,75]

* mechanistic/extrapolated evidence; not yet confirmed by dedicated MetALD-omics studies. † direct evidence from MetALD cohorts. ↕ altered profile but without a consistent unidirectional change—insufficiently supported by current evidence. ↑ and ↓ indicate an increased or reduced abundance of the compounds, respectively. MASLD and increased alcohol intake (MetALD); metabolic dysfunction-associated steatotic liver disease (MASLD); alcohol-related liver disease (ALD).

## Data Availability

No new data were created or analyzed in this study. Data sharing is not applicable to this article.

## Abbreviations

ADH: Alcohol dehydrogenase; AI: Artificial intelligence; ALD: Alcohol-related liver disease; ALT: Alanine aminotransferase; AMPK: AMP-activated protein kinase; APOA1: Apolipoprotein A1; APRI: AST to Platelet Ratio Index; AST: Aspartate aminotransferase; AUC: Area under the curve; AUDIT: Alcohol Use Disorders Identification Test; AUDIT-C: Alcohol Use Disorders Identification Test-Consumption; AUROC: Area under the receiver operating characteristic curve; BCAA: Branched-chain amino acids; BMI: Body mass index; CAP: Controlled attenuation parameter; CAR: Acylcarnitine/carnitine species; CD36: Cluster of differentiation 36; CDT: Carbohydrate-deficient transferrin; Cers: Ceramides; ChREBP: Carbohydrate-responsive element-binding protein; CI: Confidence interval; CLD: Chronic liver disease; CYP2E1: Cytochrome P450 2E1; DAGs: Diacylglycerols; DNL: De novo lipogenesis; DT: Digital twin; ELF: Enhanced Liver Fibrosis test; ER: Endoplasmic reticulum; FFAs: Free fatty acids; FIB-4: Fibrosis-4 index; FXR: Farnesoid X receptor; G3P: Glycerol-3-phosphate; GGT: Gamma-glutamyl transpeptidase; GSG: Glutamate–serine–glycine index; HCC: Hepatocellular carcinoma; HDL: High-density lipoprotein; HETEs: Hydroxyeicosatetraenoic acids; HR: Hazard ratio; HSCs: Hepatic stellate cells; iPSCs: Induced pluripotent stem cells; IR: Insulin resistance; KCs: Kupffer cells; LITMUS: Liver Investigation: Testing Marker Utility in Steatohepatitis; LPS: Lipopolysaccharides; LSM: Liver stiffness measurement; MASEF: Metabolomics-Advanced Steatohepatitis Fibrosis score; MASH: Metabolic dysfunction-associated steatohepatitis; MASLD: Metabolic dysfunction-associated steatotic liver disease; MELD: Model for End-Stage Liver Disease; MetALD: Metabolic dysfunction- and alcohol-related liver disease; ML: Machine learning; MRI: Magnetic resonance imaging; MRI-PDFF: Magnetic resonance imaging-proton density fat fraction; MS: Mass spectrometry; NADH/NAD+: Nicotinamide adenine dinucleotide reduced/oxidized ratio; NAFL: Non-alcoholic fatty liver; NAFLD: Non-alcoholic fatty liver disease; NAFLD-NO: Non-obese non-alcoholic fatty liver disease; NAFLD-Ob: Non-alcoholic fatty liver disease with obesity; NASH: Non-alcoholic steatohepatitis; NIAAA: National Institute on Alcohol Abuse and Alcoholism; NITs: Non-invasive tests; NLRP3: NOD-like receptor family pyrin domain containing 3; NMR: Nuclear magnetic resonance; PAMPs: Pathogen-associated molecular patterns; PBT: Patient biomimetic twin; PC: Phosphatidylcholine; PC-PLC: Phosphatidylcholine-specific phospholipase C; PDFF: Proton density fat fraction; PDT: Patient digital twin; PE: Phosphatidylethanolamine; PEth: Phosphatidylethanol; PNPLA3: Patatin-like phospholipase domain-containing protein 3; PPAR-α: Peroxisome proliferator-activated receptor alpha; PRO-C3: Type III collagen formation marker; PRO-C4: Type IV collagen formation marker; PRO-C6: Type VI collagen formation marker; PUFAs: Polyunsaturated fatty acids; ROS: Reactive oxygen species; SCFAs: Short-chain fatty acids; SLD: Steatotic liver disease; SREBP-1c: Sterol regulatory element-binding protein 1c; T2DM: Type 2 diabetes mellitus; TCA: Tricarboxylic acid; TG: Triglycerides; TGR5: Takeda G-protein-coupled receptor 5; TLR4: Toll-like receptor 4; TLR9: Toll-like receptor 9; TMA: Trimethylamine; TMAO: Trimethylamine-N-oxide; VLDL: Very-low-density lipoprotein.
